# Crystal structure of bis­{2-[amino(iminium­yl)meth­yl]-1,1-di­methyl­guanidine} carbonate methanol disolvate

**DOI:** 10.1107/S2056989015016771

**Published:** 2015-09-12

**Authors:** Jinlong Dong, Bin Liu, Binsheng Yang

**Affiliations:** a Institute of Molecular Science, Key Laboratory of Chemical Biology of Molecular Engineering of Education Ministry, Shanxi University, Taiyuan 030006, People’s Republic of China

**Keywords:** crystal structure, metformin, sodium carbonate, hydrogen bonding

## Abstract

In the title solvated mol­ecular salt, 2C_4_H_12_N_5_^+^·CO_3_^2−^·2CH_3_OH, the complete carbonate ion is generated by crystallographic twofold symmetry, with the C atom and one O atom lying on the rotation axis. The cation is twisted about the central C—N bond [C—N—C—N = −137.7 (6)°]. In the crystal, the components are linked by N—H⋯O, N—H⋯N and O—H⋯O hydrogen bonds, generating a three-dimensional supra­molecular network.

## Related literature   

For background to and medical applications of metformin (systematic name: *N*,*N*-di­methyl­imidodicarbonimidic di­amide), see: Castagnolo *et al.* (2011 ▸); De Jager *et al.* (2005 ▸); Pérez-Fernández *et al.* (2013 ▸); Yardımcı & Özaltın (2005 ▸); Xi *et al.* (2008 ▸); Li *et al.* (2005 ▸). For a related structure, see: Huang *et al.* (2008 ▸).
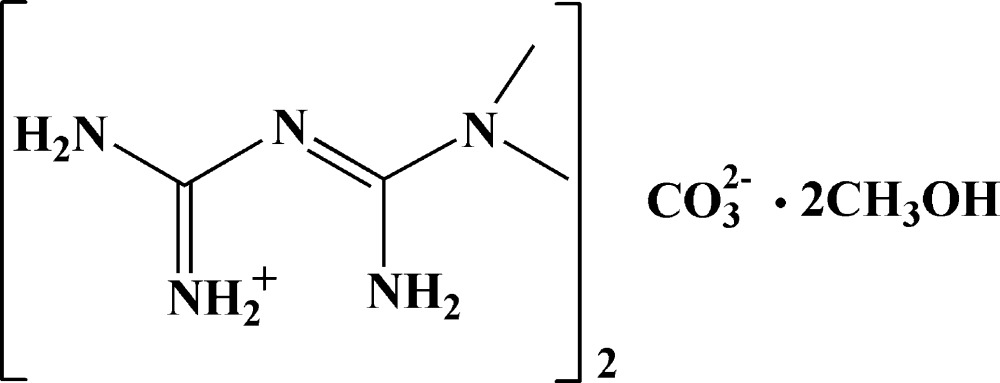


## Experimental   

### Crystal data  

2C_4_H_12_N_5_^+^·CO_3_^2−^·2CH_4_O
*M* *_r_* = 384.46
Monoclinic, 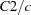

*a* = 13.5726 (12) Å
*b* = 10.5634 (8) Å
*c* = 13.9825 (13) Å
β = 90.386 (1)°
*V* = 2004.7 (3) Å^3^
*Z* = 4
Mo *K*α radiation
μ = 0.10 mm^−1^
*T* = 298 K
0.40 × 0.32 × 0.29 mm

### Data collection  

Bruker APEXII CCD diffractometer
Absorption correction: multi-scan (*SADABS*; Bruker, 2000 ▸) *T*_min_ = 0.961, *T*_max_ = 0.971
4837 measured reflections
1749 independent reflections
947 reflections with *I* > 2σ(*I*)
*R*_int_ = 0.065

### Refinement  

*R*[*F* ^2^ > 2σ(*F* ^2^)] = 0.083
*wR*(*F* ^2^) = 0.280
*S* = 1.02
1749 reflections
123 parameters
6 restraints
H-atom parameters constrained
Δρ_max_ = 0.64 e Å^−3^
Δρ_min_ = −0.29 e Å^−3^

### 

Data collection: *APEX2* (Bruker, 2000 ▸); cell refinement: *SAINT* (Bruker, 2000 ▸); data reduction: *SAINT*; program(s) used to solve structure: *SHELXS97* (Sheldrick 2008 ▸); program(s) used to refine structure: *SHELXL97* (Sheldrick, 2008 ▸); molecular graphics: *SHELXTL* (Sheldrick, 2008 ▸); software used to prepare material for publication: *SHELXTL*.

## Supplementary Material

Crystal structure: contains datablock(s) I. DOI: 10.1107/S2056989015016771/hb7495sup1.cif

Structure factors: contains datablock(s) I. DOI: 10.1107/S2056989015016771/hb7495Isup2.hkl

Supporting information file. DOI: 10.1107/S2056989015016771/hb7495Isup3.cml

**Fig. 1.**
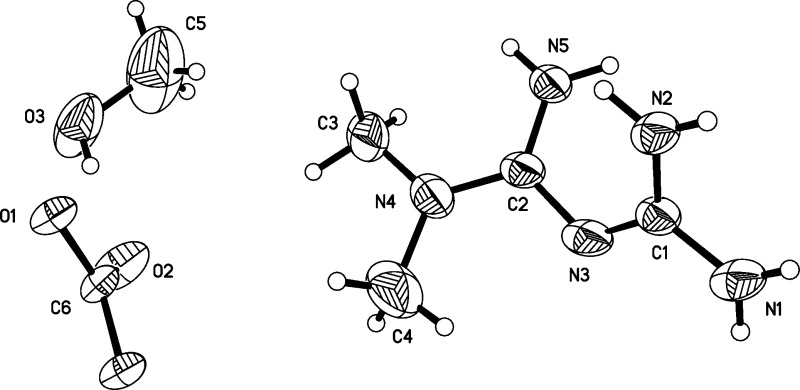
The molecular structure of the title compound with displacement ellipsoids drawn at the 30% probability level.

. DOI: 10.1107/S2056989015016771/hb7495fig1.tif
The mol­ecular structure of the title compound with displacement ellipsoids drawn at the 30% probability level.

**Fig. 2.**
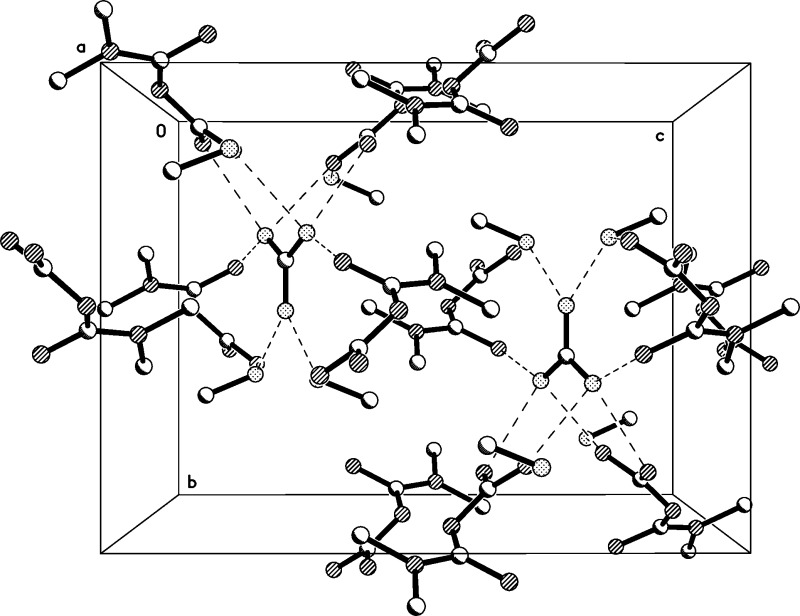
Part of the crystal structure with the hydrogen bonds drawn as dashed lines.

. DOI: 10.1107/S2056989015016771/hb7495fig2.tif
Part of the crystal structure with the hydrogen bonds drawn as dashed lines.

CCDC reference: 1422939

Additional supporting information:  crystallographic information; 3D view; checkCIF report

## Figures and Tables

**Table 1:** Hydrogen-bond geometry (, )

*D*H*A*	*D*H	H*A*	*D* *A*	*D*H*A*
N1H1*A*O1^i^	0.86	2.04	2.883(5)	166
N1H1*B*N3^ii^	0.86	2.21	3.069(6)	175
N2H2*A*O1^iii^	0.86	1.96	2.818(5)	178
N2H2*B*O3^iv^	0.86	2.08	2.896(6)	159
N5H5*A*O1^iv^	0.86	1.95	2.728(4)	150
O3H3O2	0.82	1.77	2.591(5)	177

Symmetry codes: (i) 
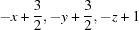
; (ii) 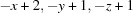; (iii) 
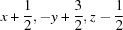
; (iv) 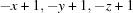.

## supplementary crystallographic information

### S1. Structural commentary

Metformin, an oral anti­diabetic drug, has been extensively used throughout the world over the last five decades to treat type-2 diabetes mellitus[Castagnolo *et al.* , 2011; De Jager *et al.* , 2005; Pérez-Fernández *et al.* , 2013 ], in particular, in overweight and obese people. Metformin has a distinct advantage of lowering serum glucose levels without causing hyper-insulinemia and subsequent risk of hypoglycemia [Yardimci *et al.* , 2005; Xi *et al.* , 2008; Liu *et al.* , 2005]. In order to find a substance that enhances the therapeutic effects of metformin, and exhibits additional pancreas-protecting effects, we synthesized the title compound (Fig. 1). Some examples of related structures already appear in the literature[Pérez-Fernández *et al.* , 2013; Huamg *et al.* , 2008]. The structure of the title compound contains two metformin molecules, one methanol molecule and carbonate ion (Fig. 1). In the crystal, N—H···O, N—H···N and O—H···O hydrogen bonds connect molecules to form a two-dimensional network parallel to (001) (Fig. 2).

### S2. Refinement details

Crystal data, data collection and structure refinement details are summarized in Table 1.The N—H hydrogen atom was located in a difference Fourier map and freely refined: N—H = 0.86 Å. The C-bound H-atoms were included in calculated positions and treated as riding atoms: C—H = 0.96 Å with *U*_iso_ (H) = 1.2 or 1.5*U*_eq_ (C).

### S3. Synthesis and crystallization

A methanol solution (20 ml) of sodium carbonate (485 mg, 3.03 mmol) and metformin hydro­chloride (500 mg, 3.03 mmol) was stirred for 12 h at room temperature. The solid part (sodium chloride) was filtered off. The rest of the solution was slowly evaporated at room temperature, yielding colourless blocks of the title compound.

### Crystal data

**Table:**

2C_4_H_12_N_5_^+^·CO_3_^2^^−^·2CH_4_O	*F*(000) = 832
*M**_r_* = 384.46	*D*_x_ = 1.274 Mg m^−^^3^
Monoclinic, *C*2/*c*	Mo *K*α radiation, λ = 0.71073 Å
*a* = 13.5726 (12) Å	Cell parameters from 1052 reflections
*b* = 10.5634 (8) Å	θ = 2.8–22.8°
*c* = 13.9825 (13) Å	µ = 0.10 mm^−^^1^
β = 90.386 (1)°	*T* = 298 K
*V* = 2004.7 (3) Å^3^	Block, colourless
*Z* = 4	0.40 × 0.32 × 0.29 mm

### Data collection

**Table:**

Bruker APEXII CCD diffractometer	947 reflections with *I* > 2σ(*I*)
Radiation source: fine-focus sealed tube	*R*_int_ = 0.065
φ and ω scans	θ_max_ = 25.1°, θ_min_ = 2.8°
Absorption correction: multi-scan (*SADABS*; Bruker, 2000)	*h* = −16→15
*T*_min_ = 0.961, *T*_max_ = 0.971	*k* = −12→11
4837 measured reflections	*l* = −16→16
1749 independent reflections	

### Refinement

**Table:**

Refinement on *F*^2^	6 restraints
Least-squares matrix: full	Hydrogen site location: inferred from neighbouring sites
*R*[*F*^2^ > 2σ(*F*^2^)] = 0.083	H-atom parameters constrained
*wR*(*F*^2^) = 0.280	*w* = 1/[σ^2^(*F*_o_^2^) + (0.1395*P*)^2^ + 3.7847*P*] where *P* = (*F*_o_^2^ + 2*F*_c_^2^)/3
*S* = 1.02	(Δ/σ)_max_ < 0.001
1749 reflections	Δρ_max_ = 0.64 e Å^−^^3^
123 parameters	Δρ_min_ = −0.29 e Å^−^^3^

### Special details

**Table:**

Geometry. All e.s.d.'s (except the e.s.d. in the dihedral angle between two l.s. planes) are estimated using the full covariance matrix. The cell e.s.d.'s are taken into account individually in the estimation of e.s.d.'s in distances, angles and torsion angles; correlations between e.s.d.'s in cell parameters are only used when they are defined by crystal symmetry. An approximate (isotropic) treatment of cell e.s.d.'s is used for estimating e.s.d.'s involving l.s. planes.

### Fractional atomic coordinates and isotropic or equivalent isotropic displacement parameters (Å^2^)

**Table:**

	*x*	*y*	*z*	*U*_iso_*/*U*_eq_	
N1	0.9937 (3)	0.6120 (4)	0.3964 (3)	0.0764 (14)	
H1A	1.0180	0.6664	0.3574	0.092*	
H1B	1.0312	0.5752	0.4377	0.092*	
N2	0.8445 (3)	0.6436 (4)	0.3289 (3)	0.0651 (12)	
H2A	0.8709	0.6975	0.2908	0.078*	
H2B	0.7824	0.6279	0.3250	0.078*	
N3	0.8641 (3)	0.5040 (4)	0.4562 (3)	0.0753 (14)	
N4	0.7163 (3)	0.4414 (5)	0.5183 (3)	0.0787 (14)	
N5	0.7482 (3)	0.3984 (4)	0.3619 (3)	0.0663 (12)	
H5A	0.6934	0.3578	0.3579	0.080*	
H5B	0.7857	0.4044	0.3128	0.080*	
O1	0.43093 (19)	0.6744 (3)	0.7084 (2)	0.0513 (9)	
O2	0.5000	0.4941 (4)	0.7500	0.0802 (17)	
O3	0.3680 (3)	0.3385 (6)	0.6850 (5)	0.143 (2)	
H3	0.4110	0.3854	0.7063	0.214*	
C1	0.8990 (3)	0.5850 (5)	0.3931 (3)	0.0584 (12)	
C2	0.7748 (3)	0.4516 (5)	0.4435 (3)	0.0627 (14)	
C3	0.6269 (4)	0.3681 (7)	0.5153 (4)	0.095 (2)	
H3A	0.5800	0.4085	0.4737	0.143*	
H3B	0.6001	0.3621	0.5785	0.143*	
H3C	0.6410	0.2847	0.4917	0.143*	
C4	0.7393 (6)	0.5018 (9)	0.6077 (5)	0.127 (3)	
H4A	0.8000	0.5476	0.6020	0.191*	
H4B	0.7458	0.4389	0.6568	0.191*	
H4C	0.6873	0.5594	0.6240	0.191*	
C5	0.3996 (9)	0.2845 (15)	0.6049 (14)	0.279 (10)	
H5D	0.3442	0.2644	0.5646	0.419*	
H5E	0.4349	0.2084	0.6203	0.419*	
H5F	0.4424	0.3419	0.5719	0.419*	
C6	0.5000	0.6130 (5)	0.7500	0.0422 (13)	

### Atomic displacement parameters (Å^2^)

**Table:**

	*U*^11^	*U*^22^	*U*^33^	*U*^12^	*U*^13^	*U*^23^
N1	0.055 (2)	0.092 (3)	0.082 (3)	−0.035 (2)	−0.022 (2)	0.034 (2)
N2	0.050 (2)	0.079 (3)	0.067 (3)	−0.0205 (19)	−0.0089 (19)	0.017 (2)
N3	0.065 (3)	0.104 (3)	0.056 (2)	−0.049 (2)	−0.0220 (19)	0.023 (2)
N4	0.076 (3)	0.108 (4)	0.051 (3)	−0.041 (3)	0.000 (2)	−0.003 (2)
N5	0.058 (2)	0.093 (3)	0.047 (2)	−0.034 (2)	−0.0063 (17)	0.000 (2)
O1	0.0389 (15)	0.0469 (17)	0.068 (2)	−0.0007 (12)	−0.0176 (13)	−0.0020 (14)
O2	0.060 (3)	0.037 (3)	0.144 (5)	0.000	−0.032 (3)	0.000
O3	0.063 (3)	0.135 (4)	0.230 (7)	−0.016 (3)	0.011 (3)	−0.097 (4)
C1	0.051 (3)	0.076 (3)	0.048 (2)	−0.024 (2)	−0.009 (2)	0.006 (2)
C2	0.059 (3)	0.079 (3)	0.050 (3)	−0.032 (2)	−0.012 (2)	0.015 (2)
C3	0.076 (4)	0.133 (5)	0.077 (4)	−0.048 (4)	0.007 (3)	0.010 (4)
C4	0.140 (6)	0.180 (8)	0.062 (4)	−0.046 (6)	0.006 (4)	−0.028 (5)
C5	0.153 (9)	0.254 (15)	0.43 (2)	−0.090 (10)	0.122 (12)	−0.223 (16)
C6	0.029 (3)	0.039 (3)	0.059 (4)	0.000	−0.007 (2)	0.000

### Geometric parameters (Å, º)

**Table:**

N1—C1	1.317 (6)	C6—O1	1.276 (4)
N1—H1A	0.8600	C6—O2	1.257 (7)
N1—H1B	0.8600	O3—C5	1.330 (13)
N2—C1	1.314 (6)	O3—H3	0.8200
N2—H2A	0.8600	C3—H3A	0.9600
N2—H2B	0.8600	C3—H3B	0.9600
N3—C1	1.319 (6)	C3—H3C	0.9600
N3—C2	1.344 (5)	C4—H4A	0.9600
N4—C2	1.321 (6)	C4—H4B	0.9600
N4—C4	1.436 (8)	C4—H4C	0.9600
N4—C3	1.440 (6)	C5—H5D	0.9600
N5—C2	1.320 (6)	C5—H5E	0.9600
N5—H5A	0.8600	C5—H5F	0.9600
N5—H5B	0.8600	C6—O1^i^	1.276 (4)
			
C1—N1—H1A	120.0	N4—C3—H3B	109.5
C1—N1—H1B	120.0	H3A—C3—H3B	109.5
H1A—N1—H1B	120.0	N4—C3—H3C	109.5
C1—N2—H2A	120.0	H3A—C3—H3C	109.5
C1—N2—H2B	120.0	H3B—C3—H3C	109.5
H2A—N2—H2B	120.0	N4—C4—H4A	109.5
C1—N3—C2	120.4 (4)	N4—C4—H4B	109.5
C2—N4—C4	121.7 (5)	H4A—C4—H4B	109.5
C2—N4—C3	122.1 (4)	N4—C4—H4C	109.5
C4—N4—C3	116.2 (5)	H4A—C4—H4C	109.5
C2—N5—H5A	120.0	H4B—C4—H4C	109.5
C2—N5—H5B	120.0	O3—C5—H5D	109.5
H5A—N5—H5B	120.0	O3—C5—H5E	109.5
C5—O3—H3	109.5	H5D—C5—H5E	109.5
N2—C1—N1	117.9 (4)	O3—C5—H5F	109.5
N2—C1—N3	124.0 (4)	H5D—C5—H5F	109.5
N1—C1—N3	118.1 (4)	H5E—C5—H5F	109.5
N5—C2—N4	119.2 (4)	O2—C6—O1^i^	120.5 (2)
N5—C2—N3	122.0 (4)	O2—C6—O1	120.5 (2)
N4—C2—N3	118.4 (4)	O1^i^—C6—O1	119.0 (5)
N4—C3—H3A	109.5		
			
C2—N3—C1—N2	17.2 (8)	C4—N4—C2—N3	9.8 (9)
C2—N3—C1—N1	−165.0 (5)	C3—N4—C2—N3	−169.3 (6)
C4—N4—C2—N5	−177.3 (7)	C1—N3—C2—N5	49.6 (8)
C3—N4—C2—N5	3.6 (9)	C1—N3—C2—N4	−137.7 (6)

Symmetry code: (i) −*x*+1, *y*, −*z*+3/2.

### Hydrogen-bond geometry (Å, º)

**Table:**

*D*—H···*A*	*D*—H	H···*A*	*D*···*A*	*D*—H···*A*
N1—H1*A*···O1^ii^	0.86	2.04	2.883 (5)	166
N1—H1*B*···N3^iii^	0.86	2.21	3.069 (6)	175
N2—H2*A*···O1^iv^	0.86	1.96	2.818 (5)	178
N2—H2*B*···O3^v^	0.86	2.08	2.896 (6)	159
N5—H5*A*···O1^v^	0.86	1.95	2.728 (4)	150
O3—H3···O2	0.82	1.77	2.591 (5)	177

Symmetry codes: (ii) −*x*+3/2, −*y*+3/2, −*z*+1; (iii) −*x*+2, −*y*+1, −*z*+1; (iv) *x*+1/2, −*y*+3/2, *z*−1/2; (v) −*x*+1, −*y*+1, −*z*+1.
